# The impact of clinical experience on working tasks and job-related stress: a survey among 1032 Norwegian GPs

**DOI:** 10.1186/s12875-022-01810-y

**Published:** 2022-08-27

**Authors:** Tor Magne Johnsen, Børge Lønnebakke Norberg, Frode Helgetun Krogh, Hanne Dahl Vonen, Linn Okkenhaug Getz, Bjarne Austad

**Affiliations:** 1 Norwegian Centre for E-Health Research (NSE), Tromsø, Norway; 2 Department of Public Health and Nursing, Norwegian University of Science and Technology (NTNU), General Practice, Research Unit, Trondheim, Norway; 3 Midtbyen Medical Centre Trondheim, Trondheim, Norway; 4 Medical Student Norwegian University of Science and Technology (NTNU), Trondheim, Norway; 5 Øya Medical Centre, Trondheim General Practice Research Unit, Trondheim, Norway

**Keywords:** General practice, clinical experience, workload, conflictual consultations, workrelatated stress

## Abstract

**Background:**

General practice is a generalist discipline fraught with complexity. For inexperienced physicians, it may be demanding to get to grips with the clinical challenges. The purpose of this article is to describe possible differences in the range of tasks between inexperienced and experienced general practitioners (GPs), and the extent to which clinical experience affects the way in which GPs perceive their daily work.

**Methods:**

An online questionnaire was sent to all regular GPs in Norway (*N* = 4784) in 2018. The study sought to document the tasks performed during a typical working day and how the GPs perceived their working situation. In this study, we compare the tasks, working situation and occurrence of potentially conflictual consultations among ‘less experienced physicians’ (≤ 5 years of experience in general practice) versus ‘more experienced physicians’ (> 5 years of experience). The findings are discussed in light of theories on development of expertise.

**Results:**

We received responses from 1032 GPs; 296 (29%) were less experienced and 735 (71%) more experienced. The two groups reported virtually the same number of consultations (19.2 vs. 20.5) and clinical problems handled (40.4 vs. 44.2) during the study day. The less experienced physicians reported a higher proportion of challenging and/or conflictual consultations, involving prescriptions for potentially addictive medication (5.7% vs. 3.1%), sickness certification (4.1% vs. 2.4%) and referral for medical investigations on weak clinical indication (8.1% vs. 5.6%). For other clinical issues there were minor or no differences. Both GP groups reported high levels of work-related stress with negative effect on self-perceived health (61.6% vs 64.6%). GPs who felt that high job demands harmed their health tended to handle a slightly higher number of medical issues per consultation and more consultations with elements of conflict.

**Conclusions and implications:**

Inexperienced GPs in Norway handle a workload comparable to that of experienced GPs, but they perceive more conflictual consultations. These findings have relevance for training and guidance of future GP specialists. Irrespective of experience, the GPs report such high levels of negative work-related stress as to indicate an acute need for organisational changes that imply a reduced workload.

## Introduction

The Regular General Practitioner (GP) Scheme was implemented in Norway in 2001 (Table 1). For many years, the scheme was considered successful, and recruitment of GPs was satisfactory [1, 2]. However, the working situation in Norwegian primary health has become characterised by increasingly extensive and complex tasks [3, 4].

**Table 1:**
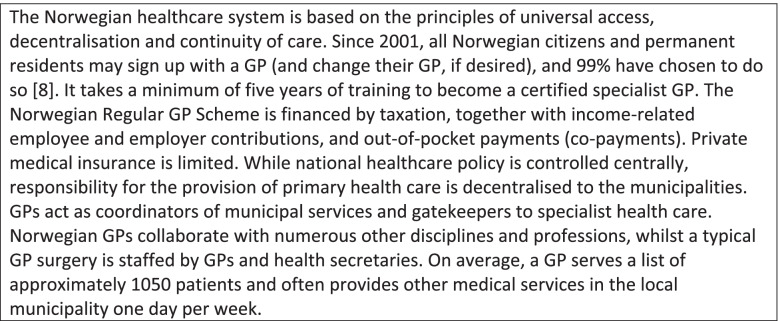
The Norwegian Regular GP Scheme

In general, GPs encounter an unselected range of people/patients and reasons for consultations. Many problems in primary care cannot be addressed by use of pre-defined algorithms or guidelines [5, 6] and only a fraction of all decisions can be categorised as genuinely ‘evidence-based’ [7]. As a result, GPs, and perhaps especially inexperienced GPs, may perceive the specialty as demanding and fraught with complexity.

The ability to handle complex problems efficiently and effortlessly generally tends to increase with experience. Various pedagogical models describe how professional knowledge and skills develop from the novice to the expert/master stage [9]. Experienced practitioners rely more on implicit and experience-based knowledge than their less experienced peers [10–13]. In light of these models, one might presume that clinical challenges would be tailored to GPs’ individual levels of competence, in terms of both complexity level and workload. Surprisingly little research has however been done on the subject [14]. We lack updated knowledge about the extent to which patient characteristics, working styles and workload differ between inexperienced and experienced doctors in regular GP schemes.

Work-related stress among physicians is a well-known phenomenon. It can be addressed by the ‘demand-control model’ [15, 16] and the ‘effort-reward imbalance model’ [16] from occupational medicine. Together, these models illustrate how a challenging but also professionally rewarding working day is characterised by a reasonable balance between challenges and perception of control and positive outcomes (sense of coping and perception of control).

Many reasons for work-related stress among GPs have been described beyond a generally fast pace of work. The complexity of general practice is challenging, and many decisions need to be made in the presence of considerable uncertainty [17–19]. On top of this, societal changes have brought increasing demands for availability and for quick and accurate answers and solutions, both from patients and collaborating actors in and beyond the healthcare system. Young Norwegian GPs who undergo specialist training are entitled to formal supervision at regular intervals, but in daily life they manage a great number of complex patients and situations single-handedly, in contrast to peer colleagues in hospital settings. Even in a group practice, the working situation of a GP can therefore appear somewhat lonely [20–27]. Since 1994, the Institute for Studies of the Medical Profession in Norway has undertaken surveys to identify the health, quality of life and working conditions of Norwegian physicians, applying the effort-reward imbalance model. A recent study showed a marked increase in the level of work-related stress among GPs in the period 2010–2019. The increase was more pronounced than among physicians in other clinical specialties [28].

A public evaluation of the Regular GP scheme in Norway in 2019 showed that only 9% of medical students and GP locums were planning a future career as GPs [22]. This entails an acute need for change to make the career path more attractive. For this purpose, we need more knowledge about the current working situation for GPs early in their career, in terms of both workload and range of tasks.

In 2018, our research group conducted an elaborate survey among GPs to address the full range of clinical activity as well as total workload. Each participating GP documented a typical working day in practice. In a previous publication based on this survey, we focused on the complex nature of many of the GPs’ working tasks [3]. The objective of this consecutive paper is to compare the workload and range of tasks between inexperienced and experienced GPs. Additionally, the study addresses the extent to which clinical experience affects the way GPs perceive their daily work, including perceived levels of unhealthy stress.

## Material and method

### Design, participants and procedure

A comprehensive, online survey in the Netigate application was designed by a project group consisting of GP clinicians and academics. The survey was distributed by email to all GPs registered in the Norwegian Medical Association (*N* = 4784) [3, 29] in the period 28 February – 8 April 2018. Completion of the survey was estimated to take 60–90 min. One reminder was sent. Only fully completed surveys were included in the analysis.

The invitation to participate in the survey contained a brief description of the content, objective and privacy safeguards, waiver and informed consent, instructions to select an ordinary, full day of practice outside out-of-hours duty, a unique link to the questionnaire and a checklist for continuous registration of activities and issues on the selected working day. The study contained demographic background variables but was otherwise anonymous. Analyses of small demographic subgroups were not performed.

The main part of the survey documented the GP’s activities on the selected working day, including all patient contacts as well as a list of administrative tasks. For each consultation, the GP recorded the number of medical issues brought up by the patient (typically 1–3 issues). Formal diagnoses were not recorded.

**Fig. 1:**
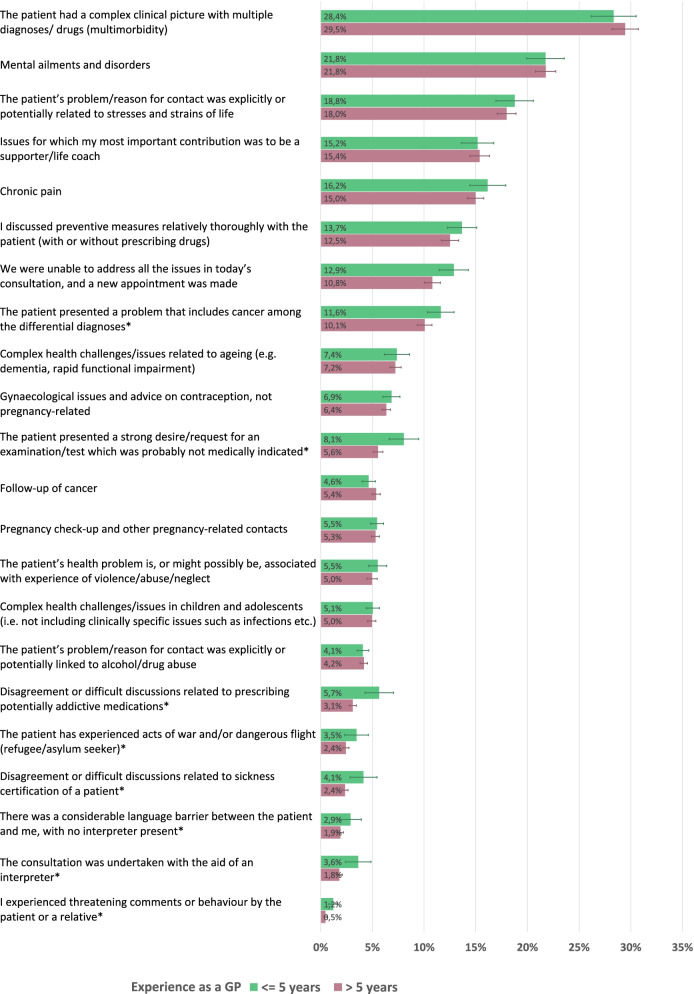
The frequency of 22 pre-defined issues during the typical working day’s consultations, divided in inexperienced (≤ 5 years) and experienced (> 5 years) GPs. Frequencies are shown as percentages of all consultations, with a 95% CI. Significant differences (p-values < 0.05) are indicated by an asterisk

In addition, the GPs registered whether the consultation was related to one or more of 22 specific topics, selected and defined by the research group as particularly relevant to document,—either due to complexity or a potential for controversy and conflict. Among these pre-defined topics were multimorbidity, chronic pain and disagreement over prescription of potentially addictive drugs. Figure 1 shows a complete list of the 22 topics.

For questions eliciting the GPs’ assessment of their work situation, response categories included ‘fully agree’, ‘partly agree’, ‘neither agree, nor disagree’, ‘partly disagree’ and ‘fully disagree’. In our subsequent analyses, we have merged the categories ‘fully/partly agree’ and ‘fully/partly disagree.’

Many of the questions used in the study were created specifically for our survey. The survey questions’ face validity was thoroughly pilot tested.

### Statistics and analysis

Descriptive data were analysed with SPSS®. We defined GPs with ≤ 5 years of work experience from general practice as ‘less experienced’, and > 5 years of work experience as ‘more experienced’. Differences between experienced and inexperienced physicians were analysed by examining the average frequency of each issue with a 95% confidence interval. In the tables and figures, the frequency of the 22 pre-defined clinical issues were reported as percentages of the average number of consultations per day. Differences in tasks/responsibilities between the groups of experience were detected by two-sided tests assuming equal variances and adjusted for all pairwise comparisons using the Bonferroni correction. Associations between the GPs’ experience of negative work-related health impact and characteristics of the registered working day were calculated with Fisher-Freeman-Halton Exact Test.

## Results

Altogether 22% (1032/4784) of Norway’s eligible regular GPs participated in the survey. Of these, 29% (296) had ≤ 5 years of experience and 71% (735) had > 5 years of experience. In total, the participating GPs registered 20 768 consultations and approximately 44 000 medical issues during the documented working days. The 296 less experienced physicians registered a total of 5700 consultations and 14 500 medical issues. Characteristics of the participating GPs are presented in Table  2. The respondents had an average of approximately 1 100 patients on their lists.

**Table 2:** Description of the total material, according to the participating GPs’ experience, age, sex, certified specialty and size of municipality

	**Experience as a GP**			
	$\le$ **5 years** ***n*** = 296		** > 5 years** ***n*** = 735	
	*n*	%	*n*	%
Total number of consultations	5676		15 092	
Average number of consultations during the day	19.2		20.5	
Average patient list size	900–999		1100–1199	
**GPs’ age (year groups)**				
< 40	264	89.2	146	19.9
40–49	28	9.5	291	39.6
50–59	3	1.0	168	22.9
60 +	1	0.3	130	17.7
**Gender**				
Female GP	163	55.1	366	49.8
Male GP	133	44.9	369	50.2
**Certified specialist**				
Specialist in general practice	15	5.1	654	89.0
GP trainee or locum	281	94.9	81	11.0
**Size of municipality (inhab.)**				
< 5000	26	8.8	54	7.3
5000 – 10 000	32	10.8	53	7.2
10 000 – 50 000	123	41.6	288	39.2
50 000 – 100 000	49	16.6	114	15.5
100 000 +	66	22.3	226	30.7

The tasks/responsibilities registered during the GPs’ selected working day are shown in Table 3. As can be seen, less experienced physicians handled almost the same number of consultations and job tasks as their more experienced colleagues.

**Table 3:** Recorded tasks/responsibilities during a typical, full working day for less experienced (≤ 5 years) and more experienced (> 5 years) GPs, respectively

Numbers of registered tasks/responsibilities during the selected *working day*	Experience as a GP		*P*-value*
	< 5 years (*n* = 296) n (95% CI)	> 5 years (*n* = 735) n (95% CI)	
Number of regular consultations	19.2 (18.6–19.7)	20.5 (20.2–20.9)	< 0.001
Total number of *issues* during the consultations	40.4 (38.5–42.3)	44.2 (43–45.5)	0.001
E-consultations	0.9 (0.7–1.2)	1.1 (1.0–1.3)	ns
Digital messages to patients	5.0 (4.4–5.5)	5.6 (5.2–6.0)	ns
Digital messaging with municipal homecare	3.8 (3.5–4.2)	4.3 (4.1–4.5)	0.032
Telephone calls with patients/relatives	3.8 (3.5–4.2)	4.4 (4.1–4.6)	0.016
Home visits	0.2 (0.1–0.3)	0.2 (0.2–0.2)	ns
Sick leave certifications issued	4.6 (4.3–4.9)	4.8 (4.6–5.0)	ns
Other certifications issued	2.8 (2.5–3.1)	3.1 (2.9–3.2)	ns
Minor surgery, proctoscopy, cryotherapy etc	1.2 (1.1–1.4)	1.2 (1.1–1.3)	ns
Gynaecological examinations	1.0 (0.8–1.1)	1.0 (0.9–1.0)	ns
Acute admissions to hospitals	0.6 (0.5–0.7)	0.5 (0.5–0.6)	ns
Referrals to specialist health services	3.4 (3.1–3.7)	3.1 (3.0–3.3)	ns
Requisitions for diagnostic imaging	2.4 (2.2–2.6)	2.2 (2.1–2.3)	ns
Discharge summaries read and signed	13.6 (12.5–14.6)	15.8 (15.1–16.5)	0.001
Medical imaging results read and signed	3.2 (3.0–3.5)	3.6 (3.4–3.8)	0.034
Drug prescriptions	23.7 (21.9–25.5)	27.5 (26.3–28.8)	0.001
Letters sent	3.0 (2.6–3.3)	3.3 (3.0–3.5)	ns

*P-values are based on two-sided tests assuming equal variances and adjusted for all pairwise comparisons using the Bonferroni correction, *ns* = not significant

Figure 1 shows the frequency of 22 pre-defined clinical issues handled by inexperienced and experienced GPs, respectively. In many cases there were only marginal differences between the two GP groups. This applied to, for example, complex multimorbidity management, present in 28.4% vs. 29.5% of the consultations, mental distress and disorders (21.8% vs. 21.8%) and situations in which physicians perceived themselves as having a central supporting role as a ‘life coach’ for the patient (15.2% vs. 15.4%). However, the inexperienced physicians more frequently reported potentially conflictual topics in their consultations. For example, ‘disagreement or difficult discussions related to prescribing of potentially addictive drugs’ were reported by 5.7% (4.3–7.1) vs. 3.1% (2.8–3.5) respectively. The inexperienced physicians also somewhat more frequently reported ‘threatening comments or behaviour by patients or relatives’ (1.2% (0.8–1.6) vs. 0.5% (0.3–0.6) of the consultations).

**Fig. 2:**
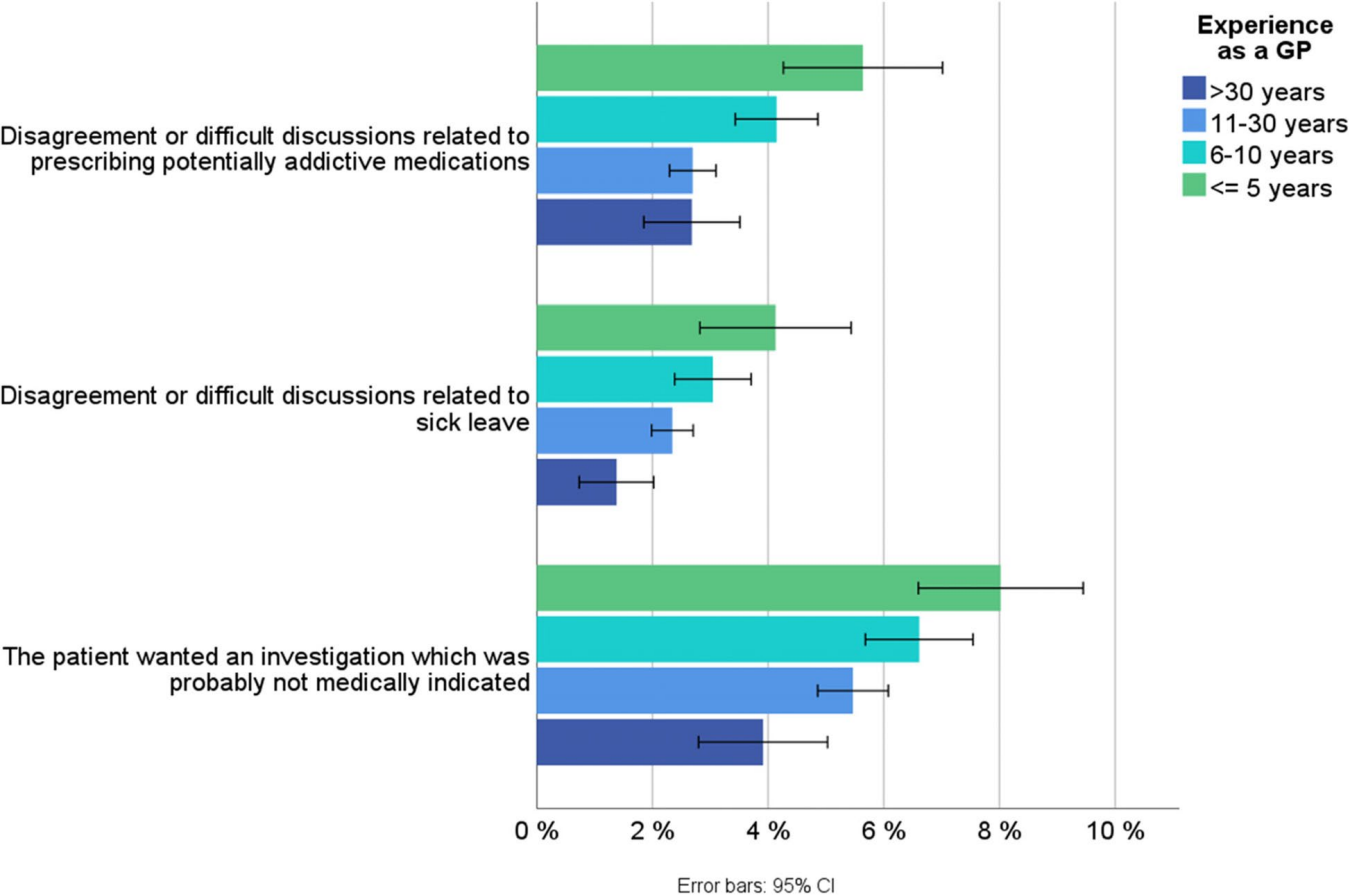
Occurrence of three potentially conflictual issues in consultations among GPs with varying degree of work experience. Frequencies are shown as percentages of all consultations, with a 95% CI

The associations between clinical experience and perception of potential conflictual consultations are shown in more detail in Fig. 2. Here, we have sub-divided the group of ‘more experienced’ (> 5 years) GPs into categories according to length of experience (6–10 years, 11–30 years and > 30 years). As can be seen, the perception of potentially conflictual issues continues to decline throughout the GP’s career.

**Fig. 3:**
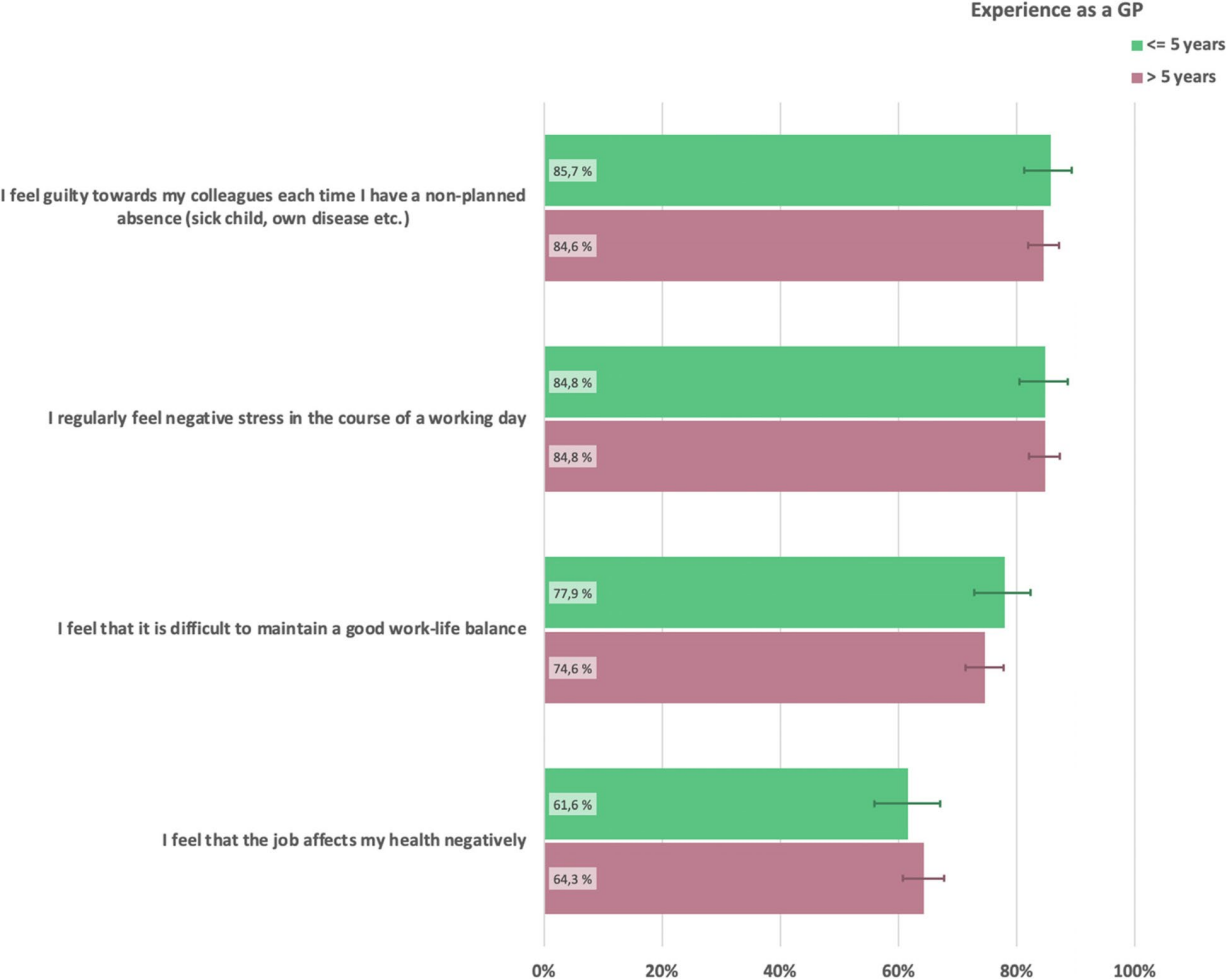
Percentage of participants who answered ‘fully or partly agree’ to the following statements about their own health, negative stress and challenges in maintaining a work-life balance among inexperienced (≤ 5 years) and experienced (> 5 years) GPs. Shown with 95% CI

The GPs’ assessment of various aspects of work-related strain and stress including subjective health impact, are shown in Fig. 3. In general, the inexperienced and experienced reported relatively similar values for the different parameters. For instance, 84% of GPs in both groups reported that they regularly experienced negative stress at work.

Table 4 shows the associations between work-related health impact and selected characteristics of the GPs’ working day. We found no direct association between the number of consultations performed and negative health impact. However, physicians who fully/partly agreed that their job affected their health negatively tended to handle a slightly higher number of medical issues and experienced more conflictual situations throughout the day compared to those who fully/partly disagreed that their job affected their health negatively. To the question of whether the job appeared to harm their health, female GPs ‘fully/partly agreed’ somewhat more frequently than males, but the difference was not statistically significant (66.7%; CI 62.5–70.7, vs. 60.3%; CI 55.9–64.5).

**Table 4:** Associations between the GPs’ experience of negative work-related health impact and characteristics of the registered working day. Shown as averages per working day with a 95% CI

	**‘I feel that my job affects my health negatively’**			***P***-values*
**Selected activities and issues**	**Fully/partly disagree** (*n* = 228)	**Neutral/Don’t know** (*n* = 138)	**Fully/partly agree** (*n* = 638)	
No. of consultations	20.1 (19.5–20.8)	19.3 (18.5–20.1)	20.4 (20.0–20.8)	P = 0.108 (0.107–0.109)
Total no. of medical issues during the consultations	40.5 (38.3–42.7)	38.8 (36.1–41.3)	45.0 (43.6–46.4)	P = 0.172 (0.171–0.173)
Disagreement or difficult discussions related to prescribing potentially addictive medications	0.5 (0.4–0.7)	0.7 (0.4–1.1)	0.9 (0.8–1.0)	P = 0.005 (0.005–0.005)
Disagreement or difficult discussions related to sick leave	0.3 (0.2–0.4)	0.5 (0.4–0.6)	0.7 (0.6–0.8)	P = 0.001 (0.001–0.001)
The patient wanted an investigation which was probably not medically indicated	0.8 (0.7–1.0)	1.1 (0.9–1.2)	1.5 (1.3–1.7)	P = 0.009 (0.009–0.009)

*P-values calculated with Fisher-Freeman-Halton Exact Test, Monte Carlo method: unbiased estimate of the exact p-value (2-sided) with a 99% confidence interval, based on 1000000 sampled tables with starting seed 205597102

## Discussion

In this study among Norwegian GPs, the main finding is that the GPs seemed to handle relatively comparable total workloads, irrespective of previous, clinical experience. The prevalence of reported negative work-related stress was high. Physicians who stated that the job impacted negatively on their health, did not have more consultations, but tended to report more consultations with elements of conflict. Compared to their more senior colleagues, inexperienced GPs reported more consultations characterised by disagreement related to sick leave certificates, requests for potentially addictive medications and seemingly unwarranted medical investigations. In total, our results indicate a demanding and hectic working life for both experienced and inexperienced GPs in Norway.

Previous research has shown that GPs’ sense of coping is affected by their own life experience, clinical experience, communication skills and familiarity with their patients over time [30, 31]. In light of the previously mentioned pedagogical models which depict the ability to analyse and deal with complex problems effectively and effortlessly as increasing in line with experience, we had expected experienced clinicians to see a higher number of patients per working day and also to report lower levels of work-related stress than their younger colleagues [9, 10]. In well-organized group practice settings, one might also presume that the senior GPs would handle a somewhat higher number of complex and demanding patients than their junior partners. None of these presumptions were confirmed by our study.

One plausible explanation for the high levels of job-related stress found in this study, is the well-documented increase in responsibilities and working hours among Norwegian GPs in recent years [22, 28, 32]. However, in combination with high workload, characteristics of general practice as a “frontline” medical discipline may also be important [33]. When assessing patients’ problems, experienced GPs may discern contributing contextual causal factors that inexperienced GPs might not recognise or focus on. However, despite experience, GPs will often lack the opportunity to mitigate detrimental psychosocial circumstances, and thus perceive an *imbalance* between the challenge at hand (a patient in need) and their actual opportunities for dealing effectively with the problem [6, 34]. In line with the previously mentioned occupational stress theories [15], a recurring feeling of professional disempowerment may under such circumstances increase the risk of poor job satisfaction and even burnout [35, 36].

GPs who stated that their job situation affected their health negatively, did not report a higher number of consultations during the day, but a tendency towards more issues handled per consultation, as well as more consultations with elements of conflict. We do not know if this reflects the original working/communication habits of these GPs, or whether persistent stress might lessen GPs’ capacity to adequately impose limits. We also acknowledge that this is probably one of several reasons why these doctors feel that their job negatively affects their health. Among other things, we cannot rule out that these doctors have longer working days which can reduce time allocated to health promoting leisure activities, with possible negative consequences for physical and mental health. Although equivocal, the results invite further discussion about the relationship between structuring of GP consultations and work-related stress.

Our findings that inexperienced GPs report more frequent conflicts with patients as well as more frequent cases of threats or violence, accord well with previous research [37, 38]. It is highly plausible that the ability to avoid conflict increases with clinical experience. However, experienced GPs may also be accustomed to a certain level of disaccord and consequently report it less frequently. GPs who generally find it hard to set limits have been shown to leave general practice and may thereby be underrepresented among experienced GPs in our material [22, 39].

### Methodological considerations

Our ambitious and time-consuming survey recruited more than 1000 GPs, corresponding to nearly one-quarter of all GPs in the Norwegian Regular GP Scheme in 2018. To achieve a higher participation rate would hardly be realistic. The demographic characteristics of the participants accord well with national GP statistics published by the Norwegian Directorate of Health [40] Physician gender and age, level of clinical experience and geographical/municipality locations were alle well represented, albeit a certain overrepresentation of younger physicians (30–39 years). In total, we consider the study sample well suited for elucidating our study's research questions.

The study was undertaken in a period of strong engagement and concerns about the sustainability of the Norwegian GP scheme [22]. The time was and is still characterized by long working hours [32], recruitment problems and smouldering dissatisfaction. It is likely to have recruited GPs who wished to help document heavy workloads and challenging clinical tasks. GPs who did not perceive their job situation as unreasonably challenging may have been somewhat less motivated to participate, and thus underrepresented. The most overburdened physicians may not have prioritised participation and may also be underrepresented in the material.

As outlined in the methods section, various questions were designed specifically for the survey. This included a question about the GPs’ subjective experience of negative work-related stress. Pilot testing indicated that this question was well understood. An ambience of collective professional concern may to some extent have coloured the GPs’ view of their working situation. Registration of specified activities and challenges during the registered day is however likely to be quite accurate [3].

## Conclusion and implications

Inexperienced and experienced Norwegian GPs reported similarly high and complex workloads during a typical working day in clinical practice, whilst the inexperienced GPs reported more potentially conflictual consultations. Furthermore, both groups reported high levels of work-related stress. In view of the recently documented reluctancy among young Norwegian physicians to choose a career in general practice, it appears crucially important to motivate and support newcomers to the field. There is acute need for organisational changes to significantly reduce the clinical workload. Furthermore, young GPs should be well supervised as they develop competence to handle complex, clinical challenges and receive good guidance on how to effectively organise their workday and consultations.

### Main message

- Inexperienced Norwegian GPs perform virtually the same volume and type of clinical tasks in the course of a typical working day as their experienced colleagues.
- Inexperienced GPs more frequently report consultations involving disagreement related to prescribing of potentially addictive drugs, requests for investigations that are not medically indicated, and sick leave certificates.
- Inexperienced and experienced GPs reported the same, high level of work-related stress.
- GPs who felt that their work was harming their health reported the same number of consultations but tended to handle more medical issues per consultation and more conflictual issues, compared to colleagues who reported lower degrees of detrimental stress.

## Data Availability

The data that support the findings of this study are available from General Practice Research Unit, Department of Public Health and Nursing, Norwegian University of Science and Technology (NTNU), but restrictions apply to the availability of these data, which were used under license for the current study, and so are not publicly available. Data are however available from the authors upon reasonable request and with permission of General Practice Research Unit, Department of Public Health and Nursing, Norwegian University of Science and Technology (NTNU).
